# Metronomic capecitabine versus best supportive care as second-line treatment in hepatocellular carcinoma: a retrospective study

**DOI:** 10.1038/srep42499

**Published:** 2017-02-13

**Authors:** Andrea Casadei Gardini, Flavia Foca, Mario Scartozzi, Nicola Silvestris, Emiliano Tamburini, Luca Faloppi, Oronzo Brunetti, Britt Rudnas, Salvatore Pisconti, Martina Valgiusti, Giorgia Marisi, Francesco Giuseppe Foschi, Giorgio Ercolani, Davide Tassinari, Stefano Cascinu, Giovanni Luca Frassineti

**Affiliations:** 1 Department of Medical Oncology, Istituto Scientifico Romagnolo per lo Studio e Cura dei Tumori (IRST) IRCCS, Meldola, Italy; 2 Unit of Biostatistics and Clinical Trials, Istituto Scientifico Romagnolo per lo Studio e la Cura dei Tumori (IRST) IRCCS, Meldola, Italy; 3 Department of Medical Oncology, University of Cagliari, Cagliari, Italy; 4 Department of Medical Oncology, National Cancer Institute “Giovanni Paolo II”, Bari, Italy; 5 Department of Medical Oncology, Rimini Hospital, Rimini, Italy; 6 Department of Onco-Ematology Medical Oncology, S.G. Moscati Hospital, Taranto, Italy; 7 Biosciences Laboratory, Istituto Scientifico Romagnolo per lo Studio e Cura dei Tumori (IRST) IRCCS, Meldola, Italy; 8 Department of Internal Medicine, Ospedale per gli Infermi, Faenza, Italy; 9 Department of General Surgery, Morgagni-Pierantoni Hospital, Forlì, Italy; 10 Department of Medical and Surgical Sciences, University of Bologna, Bologna, Italy; 11 Department of Hematology and Oncology, University of Modena and Reggio Emilia, Modena, Italy

## Abstract

Preliminary studies suggest that capecitabine may be safe and effective in HCC patients. The aim of this study was to retrospectively evaluate the safety and efficacy of metronomic capecitabine as second-line treatment. This multicentric study retrospectively analyzed data of HCC patients unresponsive or intolerant to sorafenib treatment with metronomic capecitabine or best supportive care (BSC).Median progression free survival was 3.1 months in patients treated with capecitabine (95%CI: 2.7–3.5). Median overall survival was 12.0 months (95% CI: 10.7–15.8) in patients receiving capecitabine, while 9.0 months (95% CI: 6.5–13.9) in patients receiving BSC. The result of univariate unweighted Cox regression model shows a 46% reduction in death risk for patients on capecitabine (95%CI: 0.357–0.829; *p  *=0.005) compared to patients receiving BSC alone. After weighting for potential confounders, death risk remained essentially unaltered (45%; 95%CI: 0.354–0.883; *p* = 0.013). Metronomic capecitabine seems a safe second-line treatment for HCC patients in terms of management of adverse events, showing a potential anti-tumour activity which needs further evaluation in phase III studies.

Hepatocellular carcinoma (HCC) represents the commonest primary liver cancer with increasing incidence. HCC is the 5th most widespread malignancy globally and the 3rd leading cause of cancer-related death^1^. Unfortunately most patients are diagnosed at an advanced stage when curative treatments are no longer an option.

The introduction of Sorafenib, currently representing the standard of care for advanced HCC^2^ and no proven second-line therapy is yet available for HCC patients and current guidelines recommend either best supportive care (BSC) or clinical trial enrolment^3^. According to recent studies, only 41–56% of patients failing first-line systemic therapy are potentially eligible for second-line clinical trials on the basis of clinical and biochemical eligibility criteria^4^,^5^. Capecitabine is an oral prodrug of 5-fluorouracil (5-FU), which is metabolised to 5-FU in a three-step enzymatic reaction, the last of which being the conversion in the liver and in the tumour by thymidine phosphorylase^6^. The concept of metronomic chemotherapy has been introduced in oncology in recent years^7^. Metronomic use of anti-cancer drugs can be considered as a type of “dose-dense” chemotherapy, although differing from traditional dose-dense administration. It is neither “dose-intense”, since it does not deliver more total drug per unit time, nor is it a cyclic maximum tolerated dose regimen with a three-week break period between cycles^6^. Metronomic regimens are less toxic, reporting reduced bone marrow toxicity and gastrointestinal disorders, including vomiting, nausea, mucositis and liver dysfunction. Metronomic chemotherapy was studied in different tumors^8^,^9^.

Preliminary studies suggested that capecitabine may be safe and effective in HCC patients^10^,^11^,^12^,^13^,^14^,^15^,^16^.

The aim of this study was to retrospectively evaluate the safety and efficacy of metronomic capecitabine as second-line treatment in patients who had progressed or were intolerant to first-line sorafenib.

## Patients and Methods

In this multicentric study we retrospectively analysed data of HCC patients unresponsive or intolerant to sorafenib.

Patients with advanced- or intermediate-stage HCC (either histologically proven or diagnosed according to the AASLD [American Association for the Study of Liver Diseases 2005] guidelines) unresponsive or intolerant to sorafenib, were eligible for our analysis. Sorafenib unresponsive is defined as a increase of at least 20% in the sum of the diameters of viable (enhancing) target lesions, taking as reference the smallest sum of the diameters of viable (enhancing) target lesions recorded since the treatment started or new appearance of one or more new lesions of any size.

Sorafenib intolerance is defined as CTCAE Grade ≥2 drug-related adverse event which persisted in spite of comprehensive supportive therapy according to institutional standards and persisted or recurred after sorafenib treatment interruption of at least 7 days and dose reduction by one dose level (to 400 mg once daily).

Patients treated with capecitabine received the therapy at the metronomic dosage of 500 mg every 12 h. The centers treated the patients with metronomic capecitabine whenever they were uneligible for protocol enrolment or the center had no second-line clinical trials ongoing. Eligibility criteria included: Eastern Cooperative Oncology Group (ECOG) performance status score of ≤2; Child–Pugh liver function class A or B7; adequate hematologic function (platelet count, ≥60 × 10^9^/L; hemoglobin ≥8.5 g/dL; and prothrombin time international normalized ratio ≤2.3 or prothrombin time ≤6 seconds above control]; alanine aminotransferase and aspartate aminotransferase ≤5 times the upper limit of the normal range); and adequate renal function (serum creatinine ≤1.5 times the upper limit of the normal range). Dose reductions applied when clinically indicated. Grade 3/4 adverse events (AEs) led to dose modification (500 mg daily) or temporary interruption, until symptoms resolved to grade ≤2. Follow-up consisted of a CT/MRI scan every 8 weeks or as clinically indicated. Tumor response was evaluated by modified Response Evaluation Criteria in Solid Tumors (mRECIST)^17^. Treatment with capecitabine was continued until disease progression, unacceptable toxicity or death.

Patients treated with BSC alone included patients eligible for second-line treatment (either metronomic capecitabine or clinical trial) but not complying with it. Eligibility criteria were the same as for patients treated with capecitabine.

The IRST-IRCCS-AVR Ethical Committee approved the study (approval number 1440). All patients included in the analysis were treated in accordance with the approved guidelines. Informed consent was obtained from all patients.

## Statistical analysis

Frequency tables were performed for categorical variables. Continuous variables were presented using median and range. Overall survival (OS) was defined as the time from start date of Sorafenib to date of death. AE-free patients were censored on date of last follow-up. Progression-free survival (PFS) was defined as the time from start date of capecitabine to date of progression or death or last follow-up whichever occurred first. OS and PFS were reported as median values expressed in months, with 95% confidence interval (CI). Survival curves were estimated using the product-limit method of Kaplan-Meier. The role of stratification factor was analyzed with log-rank tests. Correction for multiple testing was done as appropriate, using the Benjamini and Hochberg method.

Propensity score (PS) is the conditional probability of being treated given a set of observed potential confounders. In this way all the information from a group of potential confounders is summarized into a single balancing score variable, the so-called PS. PS assures that the distribution of measured baseline covariates is maintained unchanged in treated and untreated subjects. Standardized difference was used as balance measure to compare the difference in means in units of the pooled standard deviation.

A weighted Cox Proportional Hazard model was performed including treatment with capecitabine as covariate where all confounding factors had been controlled by weighting. PS weights were computed as 1/PS for patients treated with capecitabine and 1/(1-PS) for patients treated with BSC. Also an unweighted Cox regression model was performed.

The association between hand-foot skin reaction (HFS) and objective response (OR, defined as partial response and stable disease *vs.* progressive disease) was examined using the Chi-Square test.

*P* < 0.05 was considered statistically significant. Statistical analyses were carried out with STATA/MP 14.0 for Windows (Stata Corp LP, USA).

## Results

One hundred and thirteen consecutive patients with HCC were available for the analysis. 58 patients were treated with capecitabine from May 2011 to November 2015, and 55 patients were treated with BSC alone from December 2007 to September 2015. IRST-IRCCS recruit 38 patients treated with capecitabine and 45 patients treated with BSC. Department of Medical Oncology of Cagliari recruit 6 patients treated with capecitabine and 10 patients treated with only best supportive care. Department of Medical Oncology of Rimini recruit 5 patients treated with capecitabine. Department of Medical Oncology of the National Cancer Institute “Giovanni Paolo II” recruit 6 patients treated with capecitabine, Department of Onco-Ematology of Taranto recruit 3 patients treated with capecitabine.

Patient characteristics for the two groups are shown in Table 1. Among the capecitabine patients 44 (75.9%) were males and 14 (24.1%) females, with a median age of 67.5 years (range 37–82), while in the BSC group 42 patients (76.4%) were males and 13 (23.6%) females, with a median age of 73 years (range 28–87). Table 1 shows that patients with BSC alone and patients with capecitabine differed in age and AFP value. After application of PS, the standardized difference between patients on a second-line treatment and patients undergoing no further treatment was generally minor, which suggested that baseline characteristics between the two groups were equal (Table 2).

**Figure 1:**
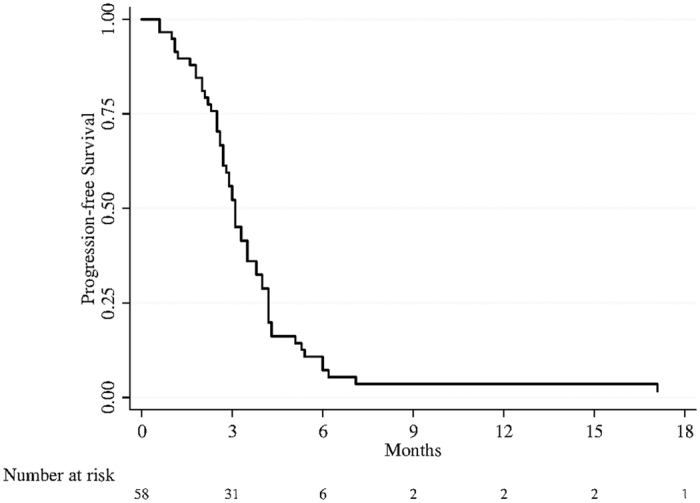
Median PFS of patients treated with capecitabine.

**Figure 2:**
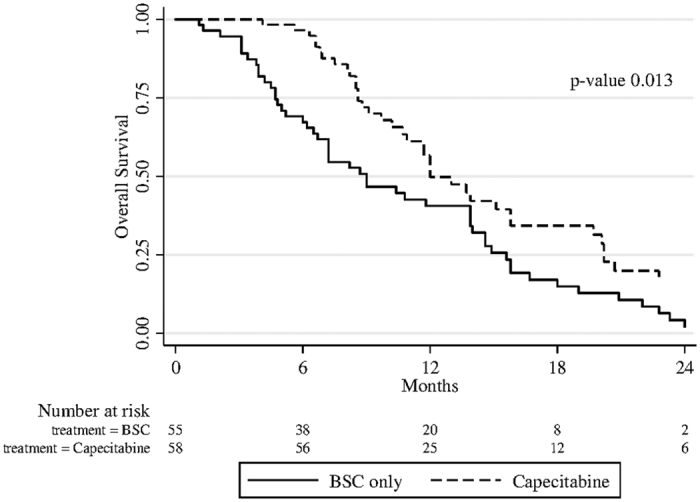
Median OS of patients treated with capecitabine and BSC only.

Median follow-up was 9 months (range 1–36 months). In patients treated with capecitabine median PFS was 3.1 months (95%CI: 2.7–3.5) (Fig. 1). Median OS was 12.0 (95% CI: 10.7–15.8) for patients receiving capecitabine, and 9.0 (95% CI: 6.5–13.9) for patients treated with BSC (Fig. 2). The result from univariate unweighted Cox regression model showed 46% reduction of death risk for patients on capecitabine (95%CI: 0.357–0.829; *p = *0.005), compared with patients on BSC alone. After weighting for potential confounders, death risk remained essentially unaltered (45%; 95%CI: 0.354–0.883; *p = *0.013).

The best tumour response in patients treated with capecitabine was partial response in 3 patients (5.4%), stable disease in 21 patients (37.5%) and progression disease in 32 patients (57,1%), according to mRECIST criteria. No complete response was observed. Twenty-three (39.7%) patients had at least one AE. The most frequent drug-related AEs were dermatologic toxicity (20.7%) and thrombocytopenia (6.9%) (Table 3).

Patients treated with capecitabine reported a significant association (*p* = 0.011) between the presence of HFS and disease control rate (Table 4).

OS with respect to patient baseline characteristics of both cohorts are also shown in Table 5. Among patients on capecitabine, better OS was reported by patients without viral infection (22.8 months [95%CI: 13.9–28.5]) than those with viral infection (10.9 months [95%CI 9.6–13.0]) (*p* = 0.0006). Among BSC patients, better OS was reported by patients with ECOG 0, meld score ≤10, LDH ≤ 220 and BCLC B. Corrections for multiple testing were made for OS in the two subgroups with unchanged results.

## Discussion

In this study we evaluated the safety and efficacy of metronomic capecitabine as second-line treatment in a cohort of HCC patients not responsive to first-line sorafenib, using a dose schedule of 500 mg twice daily. Efficacy data analysis showed that 42.9% of the capecitabine-treated patients achieved disease control, with a median PFS of 3.1 months and a median OS of 12 months. The data obtained from this study were similar to those of other studies^14^,^15^: Brandi *et al*.^16^ achieved a median PFS of 3.27 months and a median OS of 9.77 months, while Granito *et al*. achieved a median time-to-progression of 4 months and median OS of 8 months. Compared with other studies on second-line treatments, capecitabine showed median PFS longer than tivantinib (2.7 months in the high c-Met expression subgroup), yet shorter than regorafenib (4.3 months)^18^.

Unlike to other studies on capecitabine, we additionally analysed patients treated with BSC alone. The data showed a reduction in death risk for patients on capecitabine, suggesting the efficacy of the metronomic capecitabine treatment, especially in patients without viral infection and with HCV viral infection. This data supports the possible biological difference between patients with and without viral infection^19^. This finding is consistent with previous study with chemotherapy indicating that HCC correlate with infection hepatitis B have a greater aggressiveness than non-virus-related tumors^20^. HBV related tumors have different genetic mutations with greater chromosome instability respect other etiologies and have higher prevalence of loss of heterozygosity^21^. These characteristics are correlated with tumor aggressiveness and lower response to chemotherapy.

It is noteworthy that survival-predictive baseline characteristics of patients treated with BSC matched those already known from the literature (Child-Pugh score, ECOG, meld score, LDH, BCLC stage)^22^, whereas patients treated with metronomic capecitabine presented no clinical characteristics predictive of survival other than positivity or negativity to viral infection. Another interesting fact is that metronomic capecitabine might have greater effectiveness in patients with poor prognosis at baseline than in patients with better prognosis: OS was 13.9 months for BSC patients with ECOG 0 *vs.* 12.0 months for metronomic capecitabine patients; in patients with poor performance status (ECOG > 1) OS was 6.7 months for BSC treatment *vs.*13.0 months capecitabine treatment (similar to patients with ECOG 0). Our data suggested a possible activity of capecitabine in patients with poor prognosis at baseline.

Metronomic chemotherapy can also be regarded as a form of long-term ‘maintenance’ chemotherapy that can be used alone, or combined with long-term biologic targeted therapies, especially anti-angiogenic drugs such as anti-VEGFR-2 antibodies or small molecule multitargeted VEGFR-2 antagonist receptor tyrosine kinase inhibitors. A case report showed that high expression of VEGFR-2 was correlated with complete response in breast cancer patients treated with metronomic capecitabine^9^. The durable complete response to metronomic chemotherapy highlighted the importance of assessing potential predictors of benefit of this treatment, calling for further research on VEGFR2. More recently, other potential mechanisms of action have been suggested for metronomic chemotherapy involving anti-cancer immune response and other “actors” in the tumor microenvironment.

HFS is a common toxicity in patients treated with capecitabine^23^. In breast and colorectal cancer HFS was reported to be an independent predictor of treatment response to capecitabine^24^,^25^. Our data suggested that HFS may serve as an independent clinical predictor of treatment outcome.

Regorafenib in second line after sorafenib has been shown to increase survival respect to placebo^26^. Many promising drugs in phase II studies failed in subsequent phases trials, due to the faulty study design, especially for the stratification at the time of randomization, and other heterogeneous reasons, including lacking established prognostic factors after failure of sorafenib, no significant anti-tumour activity, and liver toxicity^27^,^28^.

The study has some limitations, due in particular to its retrospective nature (cases were, however, consecutively selected, thus reducing potential bias). Lack of randomization, as a technique used to balance the effect of uncontrollable factors that can impact the results of an experiment, was limited applying propensity score to baseline variables.

The results from unweighted and weighted Cox regression model showed that the reduction of death risk for patients in capecitabine group, compared with patients on BSC alone, remained essentially unaltered after weighting for potential confounders showed in Table 2, so we can conclude that baseline and first line treatment characteristics, aren’t factor so strong to change risk of death of analyzed patients.

In conclusion, metronomic capecitabine seems safe in the second-line treatment of HCC patients in terms of management of AE, showing potential anti-tumour activity, which requires further evaluation in phase III studies.

## Additional Information

**How to cite this article**: Casadei Gardini, A. *et al*. Metronomic capecitabine versus best supportive care as second-line treatment in hepatocellular carcinoma: a retrospective study. *Sci. Rep.* **7**, 42499; doi: 10.1038/srep42499 (2017).

**Publisher's note:** Springer Nature remains neutral with regard to jurisdictional claims in published maps and institutional affiliations.

## Figures and Tables

**Table 1:** Patient characteristics.

Variables	Capecitabine N (%)	BSC N (%)
Total	58	55
Sex		
Male	44 (75.9)	42 (76.4)
Female	14 (24.1)	13 (23.6)
Age, Median (range)	67.5 (37–82)	73 (28–87)
Aetiology		
Non-viral	16 (27.6)	19 (34.6)
Viral	42 (72, 4)	36 (65.4)
Viral - HBV	11 (26.2)	—
Viral - HCV	31 (73.8)	—
Child-pug score		
a	52 (89.7)	52 (94.5)
b	6 (10.3)	3 (5.5)
BCLC stage		
b	7 (12.1)	10 (18.2)
c	51 (87.9)	45 (81.8)
Performance status (ECOG)		
0	40 (69.0)	34 (61.9)
1	16 (27.6)	17 (30.9)
2	2 (3.4)	2 (3.6)
Portal hypertension		
No	44 (75.9)	35 (63.6)
Yes	14 (24.1)	20 (36.4)
Meld index, Median (range)	9 (6–15)	8 (6–15)
*missing*	*29*	*0*
AFP pre-treatment	166 (2–32, 784)	767 (1.1–43, 194)
*missing*	*8*	*6*
LDH pre-treatment	220 (98–1, 035)	252.5 (132–588)
*missing*	*26*	*15*
AFP pre-treatment, Median (range)	687 (1–46, 401)	216 (1.4–50, 000)
*missing*	*12*	*10*
LDH pre-treatment, Median (range)	230 (25–918)	227 (194–419)
*missing*	*48*	*36*

**Table 2:** Checking balance of confounders between capecitabine and BSC group after weighting.

	Mean in capecitabine	Mean in BSC	Standardized differences
Sex	0.24	0.25	−0.023
Age	63.95	65.36	−0.125
Aetiology	0.72	0.72	0.011
Child-pug score	0.10	0.06	0.157
BCLC stage	1.88	1.86	0.058
PS (ECOG)	0.31	0.32	−0.015
Portal hypertension	0.24	0.26	−0.030
Sorafenib duration	0.64	0.49	0.313
Best response to Sorafenib	0.47	0.33	0.274

**Table 3:** Toxicity.

	Total		
	Any grade N (%)	Grade 1/2 N (%)	Grade 3/4 N (%)
Overall	23 (39.7)	18 (31.0)	5 (8.6)
Hypertension	2 (3.4)	2 (3.4)	0 (0.0)
Hand-foot skin reaction	12 (20.7)	10 (17.2)	2 (3.4)
Thrombocytopenia	4 (6.9)	4 (6.9)	0 (0.0)
Edema lower limbs	2 (3.4)	2 (3.4)	0 (0.0)
Asthenia	1 (1.7)	0 (0.0)	1 (1.7)
Anemia	1 (1.7)	0 (0.0)	1 (1.7)
Heart failure	0 (0.0)	0 (0.0)	1 (1.7)

**Table 4:** Best response to capecitabine and cutaneous toxicity.

	N (%)	Cutaneous toxicity (any grade)		p-value*
		No	Yes	
PD	32 (57.1)	29 (65.9)	3 (25.0)	0.011
SD + PR	24 (42.9)	15 (34.1)	7 (75.0)	
*Not Evaluable*	*2*			

**Table 5:** OS in capecitabine and in BSC patients evaluated by log-rank test.

Variable	Capecitabine treatment (N = 58)			p-value #	No. patients (%)	BSC (N = 55)		
	No. patients (%)	No. events	Median OS (95%CI)			No. events	Median OS (95%CI)	p-value #
Age								
< = 70	35 (60.3)	27 (62.8)	12 (8.6–15.1)	0.1673	22 (40.0)	19 (37.3)	6.5 (4.2–13.9)	0.1277
>70	23 (39.7)	16 (37.2)	15.8 (9.7–22.8)		33 (60.0)	32 (62.7)	10.8 (7.2–14.6)	
Aetiology								
Non viral	16 (27.6)	8 (18.6)	22.8 (13.9–28.5)	0,0006	19 (34.6)	17 (33.3)	9.0 (6.0–13.9)	0.4124
Viral	42 (72.4)	35 (81.4)	10.9 (8.6–13.0)		36 (65.4)	34 (66.7)	8.2 (4.8–14.9)	
Aetiology II								
HBV	11 (18.9)	10 (23.3)	7.5 (5.6–10.7)		—	—	—	—
Non viral + HCV	47 (81.1)	33 (76.7)	15.1 (12.0–20.2)	0.0001	—	—	—	
Child-pugh score								
a	52 (89.6)	39 (90.7)	12.0 (10.7–15.8)	—	52 (94.5)	48 (94.1)	9.0 (7.2–13.9)	—
b	6 (10.4)	4 (9.3)	—		3 (5.5)	3 (5.9)	—	
PS ECOG								
0	40 (69.0)	30 (69.8)	12.0 (10.9–15.8)	0.9904	34 (61.8)	31 (60.8)	13.9 (7.2–15.6)	0.0063
1, 2	18 (31.0)	13 (30.2)	13.0 (8.2–22.8)		21 (38.2)	20 (39.2)	6.7 (3.9–8.2)	
Portal hypertension								
No	44 (75.9)	33 (76.7)	13.0 (10.9–19.7)	0.8785	35 (63.6)	33 (64.7)	9.0 (4.7–14.6)	0.6907
Yes	14 (24.1)	10 (23.3)	8.5 (6.8–26.3)		20 (36.4)	18 (32.3)	8.2 (6.0–14.0)	
Meld index								
< = 10	23 (79.3)	18 (75.0)	12 (8.2–20.2)	0.7075	44 (80.0)	41 (80.4)	10.8 (7.2–14.6)	0.0027
> 10	6 (20.7)	6 (25.0)	8.1 (4.1-NE)		11 (20.0)	10 (19.6)	4.7 (1.3–10.4)	
LDH								
< = 220	16 (50.0)	11 (52.4)	12 (8.8–20.1)	0.3531	15 (37.5)	14 (37.8)	15.6 (7.2–20.9)	0.0041
> 220	16 (50.0)	10 (47.6)	12 (8.5-NE)		25 (62.5)	23 (62.2)	7.2 (4.8–13.9)	
Sorafenib duration (months)								
< = 3 months	29 (50.0)	19 (44.2)	10.9 (8.8–13.7)	0.0211	42 (76.4)	39 (76.5)	7.2 (5.0–9.0)	0.0566
> 3 months	29 (50.0)	24 (55.8)	15.8 (10.7–20.2)		13 (23.6)	12 (23.5)	15.8 (13.9–19.0)	
Best overall response								
PD	31 (53.5)	24 (55.8)	9.1 (8.2–11.7)	0.0001	39 (70.9)	37 (72.5)	6.7 (4.7–9.0)	0.0004
SD + PR + CR	27 (46.5)	19 (44.2)	20.7 (13.0–25.0)		16 (29.1)	14 (27.5)	15.8 (13.9–20.9)	
Stage BCLC								
b	7 (12.1)	6 (14.0)	20.2 (6.6-NE)	0.8944	10 (18.2)	10 (19.6)	15.8 (3.8–23.3)	0.0006
c	51 (87.9)	37 (86.0)	12.0 (10.7–15.8)		45 (81.8)	41 (80.4)	7.2 (5.2–11.8)	
Pre-treatment AFP					21 (42.9)	19 (42.2)	7.2 (4.7–13.9)	0.2553
< = 400	30 (60.0)	22 (57.9)	12.0 (9.1–15.8)	0.7363	28 (57.1)	26 (57.8)	9.0 (6.2–15.8)	
> 400	20 (40.0)	16 (42.1)	10.9 (7.5–19.7)					
